# The effect of bigger human bodies on the future global calorie requirements

**DOI:** 10.1371/journal.pone.0223188

**Published:** 2019-12-04

**Authors:** Lutz Depenbusch, Stephan Klasen

**Affiliations:** 1 Faculty of Economics, University of Goettingen, Goettingen, Germany; 2 World Vegetable Center - East and Southeast Asia Regional Office, Bangkhen, Bangkok, Thailand; International Maize and Wheat Improvement center (CIMMYT), MEXICO

## Abstract

Existing studies show how population growth and rising incomes will cause a massive increase in the future global demand for food. We add to the literature by estimating the potential effect of increases in human weight, caused by rising BMI and height, on future calorie requirements. Instead of using a market based approach, the estimations are solely based on human energy requirements for maintenance of weight. We develop four different scenarios to show the effect of increases in human height and BMI. In a world where the weight per age-sex group would stay stable, we project calorie requirements to increases by 61.05 percent between 2010 and 2100. Increases in BMI and height could add another 18.73 percentage points to this. This additional increase amounts to more than the combined calorie requirements of India and Nigeria in 2010. These increases would particularly affect Sub-Saharan African countries, which will already face massively rising calorie requirements due to the high population growth. The stark regional differences call for policies that increase food access in currently economically weak regions. Such policies should shift consumption away from energy dense foods that promote overweight and obesity, to avoid the direct burden associated with these conditions and reduce the increases in required calories. Supplying insufficient calories would not solve the problem but cause malnutrition in populations with weak access to food. As malnutrition is not reducing but promoting rises in BMI levels, this might even aggravate the situation.

## Introduction

The growing demand for agricultural products is a much discussed and important topic. According to [1], the global agricultural production needs to increase on average by 1.1 percent per year between 2005/7 and 2050 to meet the increasing demand. Recent work of the EAT-Lancet commission [2] shows that by just meeting rising demand, global food systems would cause irreversible environmental damage and that a multitude of actions would be necessary to prevent this.

The United Nations formulate the goal of the global food system in the second Sustainable Development Goal (SDG) as to “End hunger, achieve food security and improved nutrition and promote sustainable agriculture”. Just looking at calories, it is well understood that current food supplies would be sufficient to feed the world population (though micro nutrient supplies are insufficient) [3]. However, an estimated 821 million persons were undernourished in 2017 [4]. This suggests that simply reacting to changes in the economic food demand will not necessarily lead to zero hunger. It has been shown that food systems will need to address the multidimensional inequality in the access to food [4] and the environmental burden of the production systems [5].

Paradoxically, while 821 million people were found to be undernourished, there also is a trend towards higher average weight at a given height, expressed in terms of the average Body Mass Index (BMI) (as estimated for the last decades by [6]). These two conditions can coexist in the same country, household or even person, in what has been described as the double burden of malnutrition [7]. While it could be thought that insufficient access to calories would reduce the trends in rising BMI and height, this is not the case. Food insecurity is not only coexisting with overweight and obesity but it is contributing to it [4]. Particularly in low- and middle-income countries, urbanization and low relative prices for energy dense but nutritionally poor foods promote the nutrition transition towards high intakes of vegetable oils, caloric sweeteners, processed grains, and animal sourced foods, that alongside reduced physical activity have been named as ultimate causes of the obesity pandemic. But while policies to stop these trends have been developed, few countries have implemented them on levels that might reverse the trend [7, 8].

### Calorie requirements in projections of current and future food systems

The work by Walpole et al. shows that rising BMI levels are not only an outcome of food systems but that they are also a major influence on it [9]. They consider that a person with a higher weight will need more energy to sustain her body functions. Quantifying this aspect on a global level, they show that a higher average weight in a population will imply a sizable increase in the nutritional energy the population would need to consume. They estimate that if adults in all countries had the same BMI distribution as the US population, the increase in energy requirements would be comparable to the requirements of 473 million adults of global average BMI in 2005. Yet, popular forecasts and policy models of global food demand do not incorporate this effect.

Studies like that of [1] or [10] are primarily based on projections of population growth, increasing incomes, associated shifts in demand (e.g. rising meat consumption), and exogenous price changes. For example, the popular third version of the International Model for Policy Analysis of Agricultural Commodities and Trade (IMPACT) [11] models changes in the representative household food demand based on income changes, food prices, prices of competing goods, the connected price and income elasticizes of demand, and changes in the household composition. By design these studies are ignorant of rising calorie requirements, as they are not following the normative goal of providing enough food to each person.

Recent papers have incorporated scenarios with exogenous calorie requirements by replacing the process in which the IMPACT model defines food demand. [5] calculates calories required by global populations to maintain a healthy BMI, assuming US population characteristics of body height and a moderately active lifestyle. The model of [2] uses a constant intake of 2500 kcal per day, about 130 kcal above the current global average estimated by [12]. These boundaries are set as an expected upper limit, allowing for variation between countries and future developments. This can be considered appropriate for international comparisons that aim to set a safe goal for global policies.

In this paper, we do not aim to challenge the results of models that estimate future demands based on economic possibilities, environmental boundaries, and health goals. Our estimates show what people would need to eat, i.e. the calories required to maintain body weights, based on different trends in BMI and height. We do not estimate what would be necessary to supply enough calories to every person to reach a healthy weight. Instead we take into account the existing trend towards greater weight, which in many countries has moved far beyond healthy levels.

To do this, we use longitudinal data on height [13] and BMI [6] to define reasonable upper bounds for future developments. The aim of the study is to show the importance of changes in calorie requirements up to the year 2100 for the modeling of agricultural and health policies. We do not use existing forecasts of future height and BMI. Previously applied methods to forecast BMI levels [14–17] are not appropriate for long-term forecasts and would add little predictive power while reducing the tractability of the scenarios. Instead we assume that countries increase their BMI at the same speed as Mexico and increase height at the Dutch speed (until reaching the BMI of Mexico and the height of Dutch in 2010). We use back-casting techniques to validate our assumptions.

We limit our estimation to calories as we can build on a literature that shows a clear relation between body weight and calorie requirements. For other macro and micro nutrients such models are not directly available, predictions would require an increasingly complex estimation, and stronger assumptions would need to be taken. This does not imply that our estimates are only addressing production needs for staple foods. First, approaches that use complex estimation systems and normative goals like [2] base the demand for different food groups on the overall calorie requirement of the average person. Second, an increased demand for calorie will further complicate efforts to produce adequate quantities of nutrient dense foods as the competition for productive resources intensifies.

## Materials and methods

### Scenarios of future population, height, and BMI

We design four scenarios to illustrate the influence of population growth, and increases in average BMI and height on calorie requirements (Table 1). We assume that these changes do not affect the demographic development, which we assume to follow the medium variant of the 2015 Revision of the UN World Population Prospects in all four scenarios. This is a clear simplification as we would expect that a connection exists (e.g. as excessive BMI correlates with increased mortality and taller height correlates with lower mortality) but simulations of these effects are not readily available. Following the World Population prospects we model calorie requirements per five-year age-sex group.

**Table 1:** Overview of the four scenarios.

	Scenario 1	Scenario 2	Scenario 3	Scenario 4
Δ BMI p.a.	Stable	Mexican trends ♂+0.37% ♀+0.57%	Stable	Mexican trends ♂+0.37% ♀+0.57%
Δ Height p.a.	Stable	Stable	Dutch trends ♂+0.071% ♀+0.079%	Dutch trends ♂+0.071% ♀+0.079%

#### Scenario 1: Population increase and stable weight

In the baseline scenario we keep the average weight per age-sex group stable. The demographic development can affect national calorie requirements not only due to a simple increase in the number of people. Changes in the balance between men and women are meaningful as in a given age group women are on average smaller and, at a given weight, their calorie requirements are below men’s. Despite having an, on average, higher BMI [6] women therefore need on average less energy than men of the same age, according to our estimation. The only exception to this rule is the population aged over 85 years in Timor-Leste. Changes in the age-composition of countries also matter as average weight, height, and physical activity differ across age groups. Lastly, the importance of a country’s average calorie requirement increases with its share of the world population.

#### Scenario 2: Increase in BMI

In this scenario we illustrate the potential influence of an increase in the average BMI in each country’s population. Between 1975 and 2014, global average BMI was estimated to have increased by 2.5 kg/m^2^ in men and 2.3 kg/m^2^ in women and the trend between 2000 and 2014 does not indicate that this development will stop in the near future [6]. Increases in BMI are inducing greater weight at a given height, thereby increasing calorie requirements.

We assume that all countries will experience an increase in average BMI of men and women as it was estimated for the Mexican population between 1975 and 2014 [6]. We choose Mexico as it is an Upper-Middle-Income country with a large population that saw a strong increase in both male and female average BMI. Mexico has high BMI levels but it is far from being an outlier. Also the speed at which the average Mexican BMI increased in absolute terms between 1975 and 2014 was surpassed by other countries. However, in this case it is surpassed by only relatively small countries (Equatorial Guinea, Saint Lucia, Samoa) [6].

Over the described time span, the male BMI in Mexico increased on average by 0.37 percent each year, reaching an average age-standardized BMI of 27.47 kg/m^2^ by 2014. Women experienced on average a growth rate of 0.57 percent per year, reaching 28.6 kg/m^2^ in 2014. Note that we ‘only’ assume that countries will reach Mexico’s level in 2010 while it could well be that the increases in BMI in these countries (as well as in Mexico) surpass the level of Mexico in 2010. Also, where the average BMI is above this level (as it is in the USA as well as in many Pacific islands) we model a decrease at the same rate (the latter being an optimistic assumption on countries’ ability to reduce overweight and obesity). So, while we model substantially rising BMI levels, the actual increase could still be much larger. At these growth rates, by 2100 all age-sex groups in all countries have the same average BMI as Mexico did in 2010.

To compare our scenario with the historical findings we calculate the global age-standardized average BMI for the world population over age 18 for the years 2010 to 2100. For the age-standardization, we follow the WHO standard as described by [18]. The average change per decade is 0.36 kg/m^2^ for men and 0.44 kg/m^2^ for women. This seems to be in a realistic range, compared to an increase by 0.63 kg/m^2^ per decade for men and 0.59 kg/m^2^ for women between 1975 and 2014 [6].

#### Scenario 3: Increase in height

Another factor increasing weight and thereby the energy requirements is an increase in the average height per person. Conditional on the health of mothers and children, this is a likely scenario which was observed over the last century in a number of countries. We choose the country with the tallest average height reached in a cohort aged 18—the Netherlands—as an example in this case. We use the average annual increase in height between 1907 and 1996 [13] to model changes in this scenario, which was 0.079 percent for Dutch women and 0.071 percent for Dutch men.

For simplification, we assume a yearly increase in average height by this factor for all cohorts between zero and 19 years of age. Therefore, growth takes place only up to age 20 in our model. Furthermore, we assume that average height does not grow beyond Dutch levels measured in 2010. As human height starts reducing in later life years we use the average factor of height reduction estimated and kindly provided by NCD Risk Factor Collaboration.

Using this method height converges towards Dutch levels by 2100 but not all groups reach it. The largest remaining differences to Dutch height, averaged over age groups, are projected for men in the Philippines, being 10.8 cm shorter, and women in Timor-Leste, missing 8.6 cm. For more information on the modeling and the convergence of heights see section 1 in S1 Appendix.

We can compare the height increase in this scenario with the historic change in the mean height of 18 year olds in each country. Over the 90 year time horizon of this paper our scenario projects the largest increases to be 11.2 cm for men (in Bahrain, Kuwait, Palestine, and Saudi Arabia) and 11.4 cm for women (in the Comoros). The median increase among countries is 8.8 cm for men and 8.1 cm for women. While these values represent a significant increase in height, they lag behind historic values. For the 100 years between 1896 and 1996 [13] estimate that the largest increases have occurred in South Korean women (20.2 cm) and Iranian men (16.5 cm). They also find that in many countries that experienced high growth rates in the early 20th century, growth rates plateaued in the last decades of the 20th century. Assuming that this indicates an upper limit to human height, lower future height increases, as in our specification, seem reasonable.

#### Scenario 4: Increase in height and BMI

In the last scenario we estimate the change in calorie requirements if height and BMI both increase in the future. This might be considered the most realistic scenario as this is what was observed in the past decades. Also, increments in average BMI are likely to go along with better nutritional status of mothers and children, thereby being correlated with an increase in the height of newborn children.

We use the same assumptions as before in calculating the increase in both factors. Yet, the rise in calorie requirements is not simply the sum of the effects of both increases. This is due to the multiplicative relation between BMI and height in the calculation of body weight.

### Data sources

Information on average BMI by age-sex group was kindly provided by NCD Risk Factor Collaboration, based on published research [6]. We made a small adjustment to the data on Algeria and Niger by assuming that the BMI of women over 85 years of age is the same as the one of women aged 80 to 84. We did this as the value for 85 year old women had extremely large confidence intervals and the point estimate were at 16.73 kg/m^2^ in Niger and at 13.59 kg/m^2^ in Algeria, which we deemed to be unrealistically low.

Regarding height, for persons under the age of 20 years we used data that was kindly made available by the FAO statistics division and which is mainly based on DHS surveys, [19], and different national surveys. Height for older age groups was calculated from data of the NCD Risk Factor Collaboration [13] with the help of the linear rate of height reduction in the population by age, which was kindly provided. The combination of the data is necessary as the second data set does not provide height data for age groups under 20 years while the data provided by FAO does not differentiate among the height of older age-groups. As we had height data for nine fewer countries than BMI data, we replaced the missing height with data from geographically and economically close countries (see Table B in S1 Appendix).

As weight rises non-linearly with height at a given BMI, we also required data on the variance of height per age-sex group in a population. We estimated this value, using data from the IFLS 5 survey of Indonesia [20]. We chose the survey as we considered Indonesia as an emerging middle income country with a large population to be a relatively good approximation of the average world population. Furthermore, in contrast to many other surveys the IFLS 5 comprises data on a comparably large population of elderly people. Using United States Data from the NHANES 2009-2010 survey [21] as a robustness check, we estimate comparable values. Averaging the difference over age groups up to age 80 the variance in heights is 0.07 cm^2^ higher for men and 0.14 cm^2^ higher for women in Indonesia compared to the USA. At these levels the differences would not have a sizable effect on our results.

Data on future demographics are taken from the medium variant of [22]. Due to missing data we merged some dependent territories into independent states. For some countries we applied data of geographically, economically or historically close countries. Table C in S1 Appendix lists these cases.

### Estimating the weight of adults in 2010

As direct measures of human weight by country are not directly available we apply a backward estimation. For adults we estimate average weight in an age-sex group from average height in meters and average BMI in kg/m^2^. The formula is derived by [9] under the necessary assumption that height and BMI are independent and the variance of height over all values of BMI is constant. Under these assumptions, the expected weight of individuals in an age-sex group *E*(*W*), can be calculated using the average BMI ($\;\bar{\;BMI\;}\;$), height ($\;\bar{\;H\;}\;$), and variance in height *V*(*H*). For the derivation of the formula and information on the sensitivity to different estimates for *V*(*H*) see section 2 in S1 Appendix.

$$E\left(W\right)=\;\bar{\;BMI\;}\;*\left(\;\bar{\;H\;}{\;}^{2}+V\left(H\right)\right)$$ (1)

Based on data availability, our age groups are divided by five year steps where for ages over 80 we have to fall back to information on younger cohorts or use aggregated estimates for 80 and above. For ages 100 and higher also population estimates are not separated in 5-year groups.

The weight of children below five years of age is based on the FAO weight for length/height tables [23] in combination with data on human height/length. We assume that the average weight in each age-sex group equals the weight of the reference population. This simplification ignores existing over- and underweight in children. The assumption has the advantage that we rule out that growth in height is inhibited by children’s underweight. This is necessary for scenarios in which we assume an increase in average height to be realistic. As underweight in children is more likely due to stunting and not wasting [24], the bias due to the overestimation of weights is likely to be small. Therefore, the overestimations of child weight in underweight populations will only partially balance out underestimations of weight in overweight populations. Hence, we are likely to systematically underestimate weight in children.

We follow the same method for the estimation of children aged five to 18. For this age group the reference standards are defined in terms of BMI and we take the reference BMI as the average BMI. As in the older age groups we use height and variance in height to deduce the average weight from the BMI value. As with the younger children, this is likely to lead to a net underestimation of weight of adolescents and thus will downward bias our resulting calorie requirements.

We compare the weight of the age-sex groups between 0 and 49 years with data from the Demographic and Health Surveys (DHS). Averaging over all countries and age groups we overestimate the weight of adult women by 122 g, and underestimate that of adult men by 96 g, suggesting a high precision of our estimate. Comparison for children is more complicated, as explained in section 3 in S1 Appendix. Averaging across all age groups, we underestimate the weight of girls aged 0 to 19 by 2.25 kg while overestimating boys’ weight by 279 g.

### Estimation of calorie requirements

Our estimations of the average calorie requirements are based on the standard formula [25]. Based on this, the calorie requirement of an adult depends on sex, age, weight and activity level of the person. We assume that on average people follow a moderately active lifestyle and use the lowest multiplier connected to this group in the standard framework [25]. Importantly, the values we estimate in this fashion are not the energy required for a person’s healthy living but the energy to maintain a person’s given weight. For healthy living it would be sufficient to provide the energy required at the lowest healthy BMI level. As a simplification, we do not take into account the energy required to build up the additional weight. Given that there are always some people gaining weight and some losing weight, a precise estimation of this factor would require to model the energy spent on increases in BMI minus the energy persons make available from energy deposits in their body. Ignoring the net energy requirement of this process introduces an underestimation in our estimations, yet we are optimistic that this effect is comparably small.

The daily calorie requirements of children above one year are based on the parameters of a quadratic regression of weight on children’s total energy expenditure and an increment for the energy required for growth [25]. We account for the energy required due to pregnancy and lactation of mothers by multiplying the number of births in each year with the estimated energy requirement for both [25]. We assume 6 months of exclusive breastfeeding and ignore miscarriages. For more details and formulas for the estimation of calorie requirements of children and adults see section 4 in S1 Appendix.

To derive the yearly calories required in a country we multiply the estimates with the population in each age-sex group, aggregate over all age-sex groups and multiply by 365. As the numbers we estimate in this way are large, we state them in tera calories (i.e. calories multiplied by 10^12^) on the country level and in exa calories (i.e. calories multiplied by 10^15^) on the global level.

## Results

**Fig 1:**
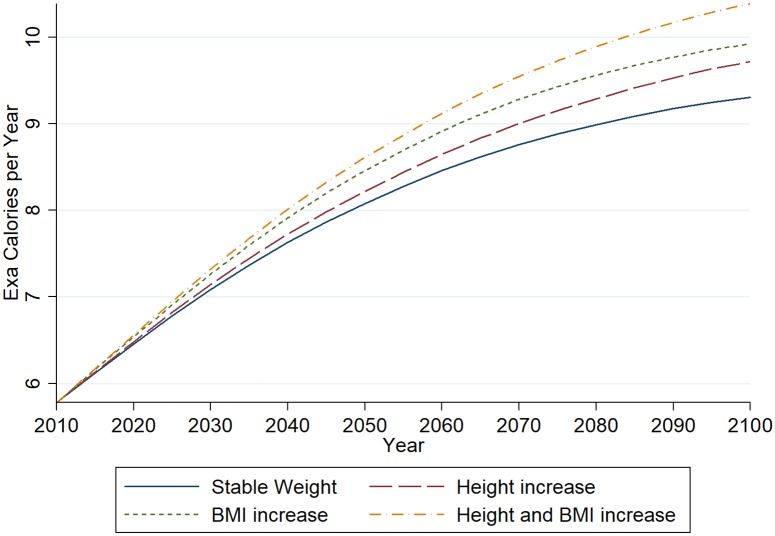
Estimations of the global requirements for calories up to the year 2100 according to the four scenarios.

Our estimations, plotted in Fig 1, show an increase in global aggregate calorie requirements that slows down over time. In the baseline scenario, holding weight per age-sex group stable, we estimate an increase in calorie requirements by 61.05 percent between 2010 and 2100. In scenario two -increase of BMI to Mexican levels- we estimate an increase by 71.72 percent. In scenario three -increase of height to Dutch levels- the value is with 68.18 percent considerably smaller. Looking at Fig 1 shows that scenario two is always associated with larger increases than scenario three, yet the difference starts to decline in the last third of the century. This reflects the much faster growth of BMI compared to height in our model. While many countries have already reached Mexican BMI levels by 2100, height is still increasing in some countries. When combining both factors in scenario four the increase is estimated to be 79.78 percent. Compared to the baseline scenario this is a 18.73 percentage points or 30.67 percent higher increase. This additional increase amounts to more than the current energy requirements of India and Nigeria combined. As expected this value is larger than the sum of increases in scenario two and three, yet with only 0.92 percentage points this difference is small.

Fig 1 further shows that increases due to demographic change are triggering particularly high growth rates in energy requirements in the first half of the 21st century. While increases due to demography level-off after this point (as population growth is estimated to slow down), the effects due to increases in height and BMI become larger. Hence, ignoring possible increases in BMI and height leads to an underestimation of the change in energy requirements, particularly in the second half of the 21st century.

As models like that of [2] base scenarios on assumptions of the average intake of kcal per capita, it is also interesting to look at these in our model. Based on our model the average daily requirement was 2285 kcal per person in 2010. With rising BMI and height, this increases to 2425 in the year 2050 and to 2538 kcal in 2100. In comparison to [2] this means that their assumption of 2500 kcal per capita is a reasonable high assumption for developments up to 2050. However, it would be overtaken by the year 2080 in scenario four.

### Changes at the country level

**Fig 2:**
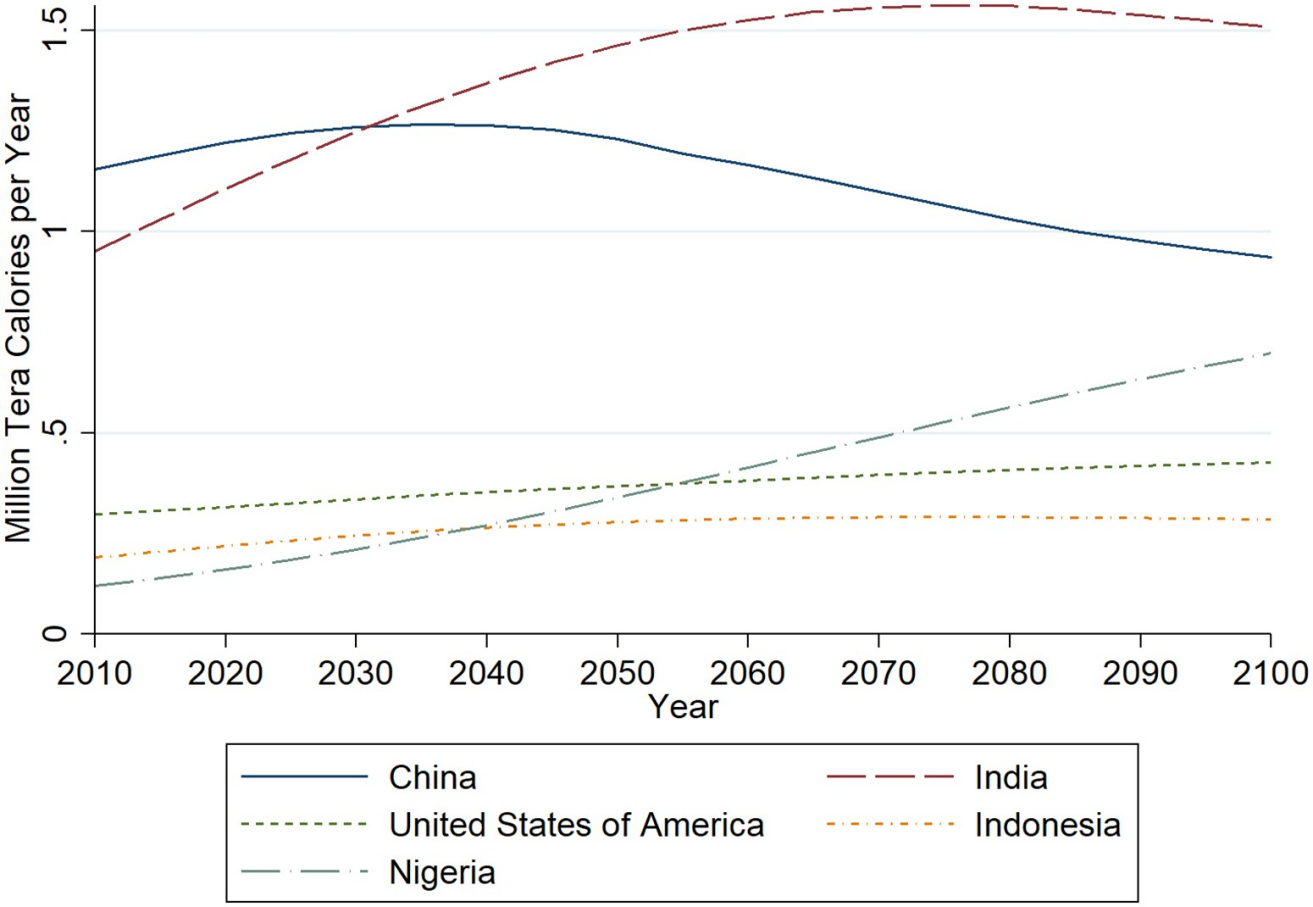
Development of calorie requirements in the four largest countries. As Nigeria joins the group and Indonesia is leaving it, predictions for both are plotted.

Disaggregating the numbers at the country level shows that not only the global requirement increases but also its geographical dispersion changes (see Table D in S1 Appendix for data on all 191 countries). We show the estimated development in the four largest countries by calorie requirements, as estimated in scenario four in Fig 2. As the fourth largest country changes from Indonesia in 2010 to Nigeria in 2100, both countries are included. First the comparison of estimates for China and India is noteworthy. China is estimated to experience an early peak in requirements before being surpassed by India. This is mainly driven by demographic developments: China will face a sharply declining population while India’s population is projected to grow much longer. Calorie requirements in the USA will peak towards the end of the century due to steady population growth, while Indonesia’s requirements will peak in the middle of the century. These countries will be surpassed by Nigeria, where rapid population growth propels requirements up.

While these movements can also be observed in scenario one (S1 Fig), the levels stay generally lower than in scenario four and the relative change amongst the countries is less intensive. In particular in scenario one the peak in Indian requirements is less pronounced and Nigeria takes over the third position later, ending up closer to US aggregate requirements.

#### Large gains across Sub-Saharan Africa and parts of the MENA region

**Fig 3:**
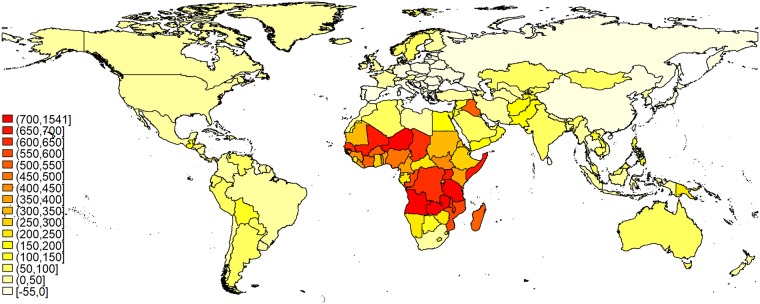
Relative change in national calorie requirements between 2010 and 2100 assuming increases in BMI and height (Scenario 4).

The estimated increase in Nigeria is typical for Sub-Saharan African countries. Overall, it is this region that experience the highest relative increases as is visible in Fig 3 (for comparison see with results from scenario one). Under scenario four, among the 22 countries for which we estimate a more then five fold increase, Iraq (with an increase by 503 percent) is the only country outside of Sub-Saharan Africa. The three countries with the largest growth rates are Niger (+1541 percent), Zambia (+833 percent), and Burundi (+725 percent).

Likewise three of the five countries with the largest absolute increases are African, as can be seen in Table 2. Putting the values in perspective: One tera calorie corresponds to the energy in 274 tons of uncooked white long-grain rice according to [26]. Therefore, the expected increase in Nigeria roughly equals the calories contained in 121 percent of mainland China’s 2010 rice harvest (milled equivalent), as estimated in the FAO food balance sheets [27].

**Table 2:** Countries with the largest absolute change in calorie requirements in Scenario 4.

Top increase		Top decrease	
Country	Tera calories	Country	Tera calories
Nigeria	+577,564	China	-218,827
India	+555,668	Japan	-29,597
DR Congo	+311,234	Russia	-21,208
Tanzania	+238,890	Thailand	-17,341
Pakistan	+201,443	Ukraine	-17,273

Southern Africa is the exception to the Sub-Saharan trend, being more comparable to the more modest increases in most of the MENA region. In that region countries with very low increases, like Morocco, Tunisia, Libya, and Turkey, exist beside Iraq, which experiences the strongest relative increase of any country outside of Africa. In between are a number of countries, including the populous Egypt, which also experiences increases by 155 percent purely due to demographics and 165 percent in the fourth scenario.

#### Moderate and negative growth rates in most of Asia, Europe, and the Americas

As Fig 3 also shows, many European countries, especially in the former Socialist regions, experience a decline in energy requirements. The only European countries with increases above 50 percent are Luxembourg, Norway and Sweden.

The negative growth rates estimated for former Socialist countries also apply to Russia and the Asian cases of China and the Caucasus, except Azerbaijan. Together with declines in Iran, Japan, Sri Lanka, Taiwan, and Thailand, changes in these countries help to dampen the expected increase in requirements in Asia. Still, strong increases in Pakistan, Afghanistan, Central Asia and parts of South East Asia can be observed at the same time. India, which due to its population size dominates absolute changes in Asia, experiences a moderate increase of 58 percent. However, in absolute numbers this modest growth translates into the second largest increase worldwide (Table 2).

Looking at the top decreases, the relative changes are clearly dominated by former socialist countries. They make up four of the top five countries in this group (Bosnia and Herzegovina, Bulgaria, Moldova, and Romania) with the exception being Taiwan. The estimated decline in these countries varies between 53 percent in Moldova and 43 percent in Taiwan. The strongest decreases in absolute numbers are dominated by China (Table 2). The comparison shows that the biggest decreases in other countries play only a small role in dampening global increases. Even the sizable decreases in China is equivalent to just 39 percent of the increase in India.

The Americas see only a relatively modest increase. A rise by more than 100 percent is only expected in Belize, Bolivia, and Guatemala. These are currently home to comparatively small populations, resulting in absolute changes well below those estimated for Africa and Asia.

### Localizing the influence of weight gains

**Fig 4:**
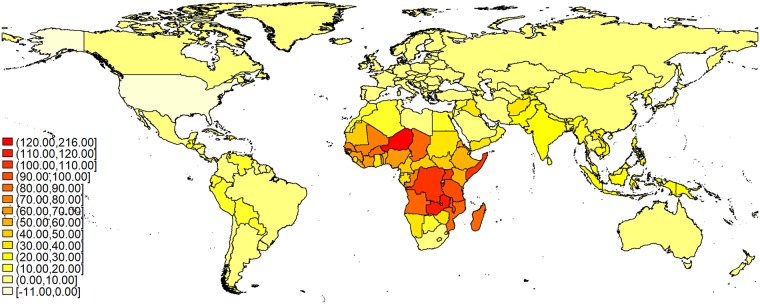
Estimated effect of BMI and height increase in Scenario 4 on energy requirements between 2010 and 2100.

Including the influence of weight gains is not only important due to its effect on world energy requirements but also as it hits some regions more than others. In order to illustrate this, we map the difference between the predicted increase between 2010 and 2100 in scenarios four and one, i.e. the additional calories that are required due to changes in BMI and height, in Fig 4.

It is clearly visible that the effect of changes in weight is stronger where we already expect the highest increases based on demographic changes (S2 Fig). In part this is due to the gains in weight being multiplied with an increasing number of people, thereby amplifying the effects of demographic change. The other factor is that especially those countries where we expect stronger population growth are characterized by comparably low height and BMI in 2010. Countries with a population that is already tall and has a high BMI in 2010 cannot increase these values much more until they reach Dutch and Mexican levels, respectively. For example, being home to a relatively tall population with a high BMI, the USA actually would need fewer calories as their BMI reduces to Mexican levels.

### Comparing required with available calories

Our estimates are about required calories for a diet that maintains the weight of everyone in the world. This is a normative concept that might bear little relation with the current actual availability of calories for human consumption. Comparing our 2010 estimates to actually available calories indicated by FAO’s food balance sheets helps to put our numbers into perspective and get an intuition of the future challenges to food security.

According to our estimates only nine countries did not have as many calories available as required. The 3-years average food deficit per person and day calculated by the FAO [28] estimates considerably larger deficits: In all countries where numbers are available this value is larger than in our calculations (see Table A in S1 Appendix) and, instead of nine, 114 countries are estimated to suffer from deficits. This difference is not only due to the usage of a three year average but to a number of conceptual differences. Most importantly, [28] takes into account the unequal distribution of food in each country, so that many countries with adequate average calorie availability will still have many people with access to too few calories. Besides this point, the main differences to our approach are that the FAO calculates calorie requirements at the lowest healthy BMI and a lower physical activity level than we use.

Inequality not only affects current needs but will also have an effect on the necessary increase in the available calories to meet future requirements. The extent of this influence will depend on a number of factors (e.g. income levels and distribution, food prices, infrastructure) and is beyond the scope of this paper. Similarly, substantially more calories will need to be produced if the current trend of rising meat consumption continues (as grain calories fed to animals generate meat and dairy products with many fewer calories), food waste increases, or more calories are used to produce non-food products such as biofuels.

## Testing assumed BMI and height trends on historical data

Any approach that uses historical trends to make a forecast of the future suffers from the necessary assumption that the underlying historic trends will persist. It is also the case that we cannot test if our assumption holds. We can however test if the same approach would have led to reasonable results if it had been done in the past.

The time span for which we can apply this approach is limited by data availability. In our case we require sufficient prior years to estimate the prevailing body seize, historical trends, and detailed demographic information. We therefore estimate changes from 1990 to 2010 and compare them to the actual values.

For the height increases we once again use trends observed in the Netherlands, which already at that point had a very tall population. For BMI trends, we do not rely on Mexico, as the strong increase in the country’s average BMI did not materialize at that point. Instead we use the trend observed in the US and also set the 1990 BMI value per age-sex group in the US as maximum.

The results of this exercise in Table 3 show that the actual global development lies between the second scenario (rising BMI) and the fourth (rising BMI and height). Scenario four delivers the closest estimates, being 0.2 percentage points, or 9 peta calories too high. This is the approximate requirement of Chad (8.87) in 2010. Based on this limited deviation, we are confident that our approach delivers reasonable bounds for future developments.

**Table 3:** Backcasting global changes between 1990 and 2010.

	Actual change	Scenario 2	Scenario 3	Scenario 4
Relative changes	+37.0%	+36.2%	+34.9%	+37.2%
Change in annual peta calories	+1560	+1527	+1471	+1571

## Discussion

Our estimates show that changes in body weight considerably add to the expected increase in future national and global calorie requirements. This will be particularly important in the second half of the 21st century, when increases due to demographic change start leveling off. The additional increases will especially affect those regions where demographic change already leads to markedly higher energy requirements. We therefore add an additional reason to stop global trends towards overweight and obesity, apart from the negative health effects (e.g. [29]).

While we focused on energy requirements in this paper, a larger concern are the future needs for food production and distribution necessary to ensure food security for all. Energy requirements are only one factor affecting food demand, though. Three other distinctive factors will have a major role: First, a considerable share of food items is lost or wasted. Agricultural production would therefore need to produce additional calories to make up for the losses. The extent of this will depend heavily on production systems, organization of the supply chain, and consumption patterns, as visible in the current variation in food loss [30].

Second, the consumption of meat and dairy products acts as a multiplier on the demand for agricultural goods as producing these goods needs additional energy in the form of feeds. Predictions on future demand for meat foresee slower growth rates around 1.3 percent per annum between 2005/2007 and 2050, not least because many countries already reached fairly high meat consumption levels [1]. It is unclear how exactly increased calorie consumption due to rising weights would affect the demand for meat and dairy products. One indication is that protein requirements rise with the amount of lean tissue in a body [31]. If meat and dairy products are used to cover this requirement, weight increases will raise the demand for meat and dairy products. Yet, dietary patterns are not necessarily time persistent and price effects will play an important role in the determination of demanded quantities vis-à-vis other protein sources.

Third, inequalities in the distribution will increase the amount of food needed to ensure zero hunger. The importance of this factor was discussed in the previous section.

When comparing our results to other estimations on future developments in food security (e.g. [1] or [2]), it is important to distinguish exactly which goal should be reached. Our calculations relate to the calories that a person needs to sustain a given body weight. Setting this as a goal requires a particular normative decision. If the goal is to ensure the capability of every person to make free decisions on their nutrition, our estimations might be a useful reference point.

Still, even when not making this normative decision, our results show that rising human weights will have a sizable effect on what people most likely would like to eat. In economic terms this relates to a higher preference for food compared to other goods. This would translate to an upward shift in the demand for food and ultimately an increase in food prices at any given level of supply. This puts an even heavier burden on countries that will already have trouble meeting requirements irrespective of this factor.

Considering the modest effect of weight increases on calorie requirements in countries with smaller population growth, the problem could at least partially be solved via trade. But for countries with rapidly increasing requirements, this would require them to have sufficient purchasing power. Given that the increase in food requirements is especially high in regions that are currently suffering from low levels of economic development, ensuring a sufficient supply of calories is likely to depend on substantial economic growth in these countries or some form of international redistribution such as massive amounts of income or food aid.

Investments in more productive food systems will be necessary but they need to be carefully balanced. Relative prices need to discourage the over-consumption of energy dense foods associated with rising BMIs. The primary focus needs to be on ensuring affordable prices of products like vegetables, fruits, pulses, and course grains that are rich in nutrients and put less pressure on the environment.

One may also argue that in those countries that are unable to secure enough calories to feed their growing populations, not gaining weight or increasing height will be one of the responses to partly address this problem. But such adjustments are rarely smooth and equitable. A more likely scenario would be that this would be accompanied by starvation for many, often affecting the most vulnerable groups the most.

## Supporting information

S1 Appendix Supplementary information. Additional information and tables on estimation methods, tested assumptions, and country level estimates.
(PDF)

S1 Fig Development of calorie requirements in the four largest countries by the same metric over time in the baseline scenario. (TIF)

S2 Fig Relative change in national calorie requirements between 2010 and 2100 assuming stable average weight in each age-sex group. (TIF)
