# Supplementary material for: The effect of bigger human bodies on the future global calorie requirements
Source: PLoS One. 2019 Dec 4;14(12):e0223188. doi: 10.1371/journal.pone.0223188 (PMC6892500; doi:10.1371/journal.pone.0223188)
Supplement: S1 Appendix — Additional information and tables on estimation methods, tested assumptions, and country level estimates. (PDF) [file pone.0223188.s001.pdf]

The effect of bigger human bodies on future global calorie requirements
S1 Appendix - Supplementary information and tables

October 22, 2019

1 Supplementary information on the modeled changes in height

We model a process in which age is inherited by the next older group after five years and the reduction in height due to five years of aging is then deducted for all age groups over 19 years. The exception is the group of 20 to 24 year olds. As the aging factor that was provided applies for all people above age 18, persons moving into the age group 20 to 24 did only experience this effect for on average 3 years. Comparing the factor of height reduction provided by NCD Risk Factor Collaboration with evidence from the Baltimore Longitudinal Study of Aging [32] shows comparable reductions in height. However, we do not use the factor from the latter study, as it does not take into account the higher mortality of smaller persons while this influence is included in the estimate of the NCD Risk Factor Collaboration.

2 Derivation of the formula for estimation of weights of adults in 2010 and sensitivity to variance estimates

In their derivation of the average weight in an age-sex group W, [9] take into account that in a population each individual deviates from the average with regard to their BMI and height. So they define the difference between average BMI ($\overline{BMI}$) and individual BMI (BMI) as $b = (BMI - \overline{BMI})$. Equivalently they define the difference between average height ($\overline{H}$) and individual height (H) as $h = (H - \overline{H})$. Therefore a group of individuals' expected weight $E(W)$ can be estimated by:

$$\begin{aligned}
E(W) &= E(BMI * H^2) = E((\overline{BMI} + b) * (\overline{H} + h)^2) \\
&= E((\overline{BMI} + b) * (\overline{H}^2 + h^2 + 2\overline{H}h)) \\
&= E(\overline{H}^2\overline{BMI} + h^2\overline{BMI} + 2\overline{H}h\overline{BMI} + \overline{H}^2 b + h^2 b + \\
&2\overline{H}^2 hb) \\
&= \overline{H}^2\overline{BMI} + E(h^2)\overline{BMI} + E(h)2\overline{H}\overline{BMI} + E(b)\overline{H}^2 + \\
&E(bh^2) + E(hb)2\overline{H} \\
&= \overline{H}^2\overline{BMI} + E(h^2)\overline{BMI} + E(bh^2) + E(hb)2\overline{H}
\end{aligned} \quad (1)$$

As we do not have data on variance in height, $V(H)$, per age-sex group for every country, we use the estimate derived for Indonesia as a general approximation. As described, the estimates for this value are not differing much between the USA and Indonesia. Considering the difference in the 2010 average BMI levels between the USA (24.79 for women and 25.43 for men) and Indonesia (21.06 for women and 20.53 for men), also the assumption that the variance in height is constant over BMI does not seem to introduce sizable biases. In fact, the difference in average variance in women's height between the USA and Indonesia of 0.14 cm^2 corresponds to a 35 g difference in weight for a population with average BMI 25.

3 Comparison of estimated weights with DHS data

As a quality and robustness check, we use direct information on weight gathered in Demographic and Health Surveys (DHS). We use the newest DHS data available in each of the age groups 20 to 29, 30 to 39, and 40 to 49 and compare it to the population averaged weight we estimated for these age groups. For women we can compare the values in 56 countries. Averaging over all countries and taking into account the relative population of each group, we overestimate weight by 122 g. In this number over and underestimations cancel each other out, though. The mean of absolute values per country does not suffer from this problem and also this is only 845 g. The largest difference we observe is 2.59 kg. For men we can only compare the values in 17 countries. On average, we underestimate male weight by 96 g with the average deviation from the measured value being 665 g. Therefore, while there are some discrepancies there is no strong deviation of the estimates in one direction: While we slightly overestimate weight in women, we slightly underestimate weight in men in the countries where we have direct observations at our disposal.

We do the same exercise for the weight of children. We can compare the weight of children aged 0 to 4 years in 56 countries, that of those aged 5 to 9 years in 53 countries for girls and 52 for boys, and that of the 10 to 19 year old in 56 countries for girls and in 19 countries for boys. Doing this we create simple weighted averages for the age groups 0 to 4, 5 to 9, and 10 to 19 years from the estimates which are available for each year of age. As we do not have information on the number of persons for every year of age we cannot construct a population averaged measure. Therefore, we ignore age differences in mortality and the potentially larger number of births in younger cohorts related to the form of the age distribution in most countries. This introduces an upwards bias into our results (as without weighting we imply that there are as many older as younger children in each age group). The comparison shows that our estimates for the age group 0 to 4 years are very close to the measured data. On average we underestimate the weight of girls by 213 g and that of boys by 142 g (with an average deviation from the real value being 510 g and 471 g respectively). For the group aged 5 to 9 years we overestimate the weight in all countries. On average girls are 5.05 kg and boys 6.1 kg too heavy in our estimates (with average deviations being identical). In the group aged 10 to 19 years we underestimate the weight of women in all and that of men in 17 out of 19 countries. On average we underestimate girls' weight by 8.08 kg and that of boys by 4.6 kg. Yet, over all large parts of the differences cancel out when we look at the population weighted average over the three age groups. In the 53 countries for which we have data for girls in all three age groups we underestimate girls' weight by on average 2.25 kg. In the 17 countries with data for boys we overestimate their weight on average by just 279 g.

4 Details on the estimation of calorie requirements

According to [25], the energy needed by an adult can be estimated given the person's sex, age, weight, and activity level. The first three factors define the basic metabolic rate of a person

(BMR). This is the energy required by a person that is awake, in a supine position, did not eat for ten to 12 hours, physically rested in the eight hours before, is mentally relaxed, and is in an environment where s/he neither needs to generate body heat nor need to cool down the body temperature. We present these values in Table E.

E: Equations for the estimation of BMR from body weight

Age *Years*	Males *BMR: kcal/day*	Females *BMR: kcal/day*
18-30	$15.057 * kg + 692.2$	$14.818 * kg + 486.6$
30-60	$11.472 * kg + 873.1$	$8.126 * kg + 845.6$
≥ 60	$11.711 * kg + 587.7$	$9.082 * kg + 658.5$

Source: [25].

The physical activity level (PAL) works as a multiplier of the BMR. We assume that on average people follow a moderately active lifestyle and use the lowest multiplier connected to this group in the frame work of [25], which is 1.7. We thereby decide for a higher PAL than is being used by the FAO in estimation of food insufficiency, which is 1.55 [28]. This difference is due to the different goals of the estimations. While [28] estimate the minimum necessary for good health, we estimate what is most likely metabolized in everyday life. I.e. the lower PAL is chosen by the FAO to relate to the minimum under which healthy body functions can be sustained. Our estimates are not supposed to relate to the lower bound but to the likely average requirements of a population. Notice that as the PAL acts as a multiplier, increases in the BMR due to higher body weight will have a more than proportional effect on the calorie requirement.

As a simplification, we do not take into account the energy required to build up the additional weight that comes with gains in BMI and height. Given that there are always some people gaining weight and some losing weight, a precise estimation of this factor would require to model the energy spent on increases in BMI minus the energy persons make available from energy deposits in their body. Ignoring the net energy requirement of this process introduces an underestimation in our estimations, yet we are optimistic that this effect is comparably small.

Denoting the calories used for growth as ι_f for girls and ι_m for boys, the formula for children's calorie requirements are given for girls as

$$kcal/day_f = 263.4 + 65.3 * kg - 0.454 * kg^2 + \iota_f \tag{2}$$

and for boys as

$$kcal/day_m = 310.2 + 63.3 * kg - 0.263 * kg^2 + \iota_m. \tag{3}$$

These estimations already account for energy spend on activities.

For children not older than 12 months we use another estimate by [25], specifying the calorie requirements as

$$kcal/day = -95.4 + 88.3 * kg + \iota_i. \tag{4}$$

Also here ι_i defines the increment for growth, but there is no differentiation between boys and girls requirements. As we also include the energy requirements for lactation, we only estimate the energy necessary in the seventh to 12th month. The increment for growth is the average

requirement over the respective month. As we do not have height data per month, we have to use the average height of all infants, thereby including the height of those six month and younger. Thereby, we underestimate the actual height of the seven to 12 month olds, which again leads to an underestimation of calorie requirements in this age group. This bias is (partially) reduced by a second bias: As we only have data on the total population aged zero to four years, we assume that the population is evenly distributed over age in this group. This is most likely not the case as due to child mortality the number of children reduces over age and in a growing population younger cohorts are larger. Hence, we underestimate the share of below one year olds among the population of the zero to four years old.

References

[32] Sorkin JD, Muller DC, Andres R. Longitudinal Change in Height of Men and Women: Implications for Interpretation of the Body Mass Index: The Baltimore Longitudinal Study of Aging. American Journal of Epidemiology. 1999;150(9):969–977.

A: **Calorie deficits in 2010 and comparison to FAO figures.** Comparison of estimated average per capita and day calorie requirements, with calories available for human consumption in 2010, the difference between the two, and the three years average food deficit in kcal per capita and day. For Antigua and Barbuda and Grenada no information on the last point is available.

Country	Available kcal (avg. p.c. & day)	Required kcal (avg p.c. & day)	Surplus kcal (avg p.c. & day)	FAO food deficit kcal (3-year avg p.c. & day)
Antigua and Barbuda	2316	2443	-127	
Bolivia	2177	2218	-41	182
Botswana	2234	2282	-48	227
Grenada	2432	2440	-8	
Haiti	2169	2233	-64	503
Namibia	2055	2201	-146	278
North Korea	2089	2247	-158	341
Tajikistan	2105	2267	-162	292
Zambia	1904	2030	-126	442

B: Missing data on height and their replacements.

Country	Replaced with data from
Andorra	Spain
Bahrain	Saudi Arabia
Bhutan	India
Equatorial Guinea	Sao Thome & Principe
Micronesia, Federated States of	Vanuatu
Puerto Rico	USA
Qatar	UAE
Singapore	South Korea
Tonga	Vanuatu

C: **Countries and territories with missing data on BMI or completely missing data.** Next to it are the countries that where used to replace the information or into which the population was merged.

Country	Replaced with data from	Merged into
Aruba	Saint Kitts and Nevis	
Curacao	Netherlands	
French Guyana		France
Guadeloupe		France
Guam		USA
Macao		PRC
Martinique		France
Mayotte		France
New Caledonia		France
Reunion		France
South Sudan	Sudan	
US Virgin Islands		USA
West Sahara	Morocco	

D: **Country/territory level results under all four scenarios.** The numbers display the relative increase in calorie requirements between 2010 and 2100. Each column shows the result for one of the four scenarios.

Country	Stable Weight pct.change	Rising BMI pct. change	Rising Height pct. change	Rising Height & BMI pct. change
Afghanistan	127	135	149	158
Albania	-41	-38	-40	-37
Algeria	70	76	79	85
Angola	627	660	681	717
Antigua and Barbuda	31	38	31	38
Argentina	44	51	45	51
Armenia	-40	-38	-39	-36
Aruba	-18	-13	-19	-15
Australia	89	93	90	94
Austria	-3	-1	0	2
Azerbaijan	5	10	6	12
Bahamas	37	44	36	44
Bahrain	20	27	25	32
Bangladesh	11	16	24	30
Barbados	-9	-6	-8	-5
Belarus	-29	-27	-27	-26
Belgium	19	22	21	24
Belize	120	128	119	128
Benin	305	325	337	359
Bhutan	9	14	17	23
Bolivia	89	99	96	107
Bosnia and Herzegovina	-52	-51	-50	-50
Botswana	86	93	97	105
Brazil	0	5	3	8
Brunei	22	31	25	35
Bulgaria	-55	-54	-54	-53
Burkina Faso	463	488	517	546
Burundi	609	642	687	725
Cabo Verde	40	45	49	54
Cambodia	67	77	86	97
Cameroon	332	351	359	380
Canada	43	47	43	47
Central African Republic	200	215	228	246
Chad	542	567	603	632
Chile	14	21	14	21
China	-28	-24	-23	-19
China (Hong Kong SAR)	8	13	14	21
Colombia	-1	3	1	6
Comoros	250	269	274	295
Congo	477	505	523	555
Costa Rica	8	15	10	17
Cote d'Ivoire	437	464	473	503
Croatia	-40	-39	-39	-38

D: **Country/territory level results under all four scenarios.** The numbers display the relative increase in calorie requirements between 2010 and 2100. Each column shows the result for one of the four scenarios.

Country	Stable Weight pct.change	Rising BMI pct. change	Rising Height pct. change	Rising Height & BMI pct. change
Cuba	-39	-37	-37	-34
Curacao	41	41	44	44
Cyprus	23	29	22	28
Czech Republic	-18	-16	-18	-17
DR Congo	546	575	614	649
Denmark	22	23	26	27
Djibouti	39	47	50	59
Dominican Republic	23	30	27	34
Ecuador	67	75	70	79
Egypt	155	168	151	165
El Salvador	-26	-22	-25	-21
Equatorial Guinea	335	357	357	382
Eritrea	256	271	300	319
Estonia	-33	-32	-32	-31
Ethiopia	195	209	235	251
Fiji	-18	-15	-17	-14
Finland	8	10	9	11
France	21	25	25	28
French Polynesia	8	11	6	8
Gabon	206	220	221	236
Gambia	477	507	518	552
Georgia	-43	-41	-43	-41
Germany	-23	-22	-22	-21
Ghana	218	234	239	257
Greece	-35	-33	-36	-33
Grenada	-31	-29	-29	-27
Guatemala	151	162	159	171
Guinea	379	402	420	446
Guinea Bissau	259	276	288	308
Guyana	-19	-15	-16	-13
Haiti	40	46	50	57
Honduras	46	52	51	58
Hungary	-36	-34	-35	-33
Iceland	20	21	22	23
India	36	42	51	58
Indonesia	29	37	41	50
Iran	-8	-3	-5	0
Iraq	472	501	474	503
Ireland	38	41	37	41
Israel	141	147	142	148
Italy	-18	-15	-17	-14
Jamaica	-38	-35	-36	-34
Japan	-36	-34	-31	-28

D: **Country/territory level results under all four scenarios.** The numbers display the relative increase in calorie requirements between 2010 and 2100. Each column shows the result for one of the four scenarios.

Country	Stable Weight pct.change	Rising BMI pct. change	Rising Height pct. change	Rising Height & BMI pct. change
Jordan	132	143	127	138
Kazakhstan	54	62	56	64
Kenya	320	335	356	372
Kiribati	151	158	146	153
Kuwait	110	118	105	113
Kyrgyzstan	71	80	75	84
Laos	68	84	84	103
Latvia	-40	-39	-39	-38
Lebanon	8	12	7	12
Lesotho	85	94	96	106
Liberia	335	361	364	393
Libya	33	38	33	38
Lithuania	-36	-35	-36	-35
Luxembourg	100	104	101	105
Macedonia (TFYR)	-30	-28	-28	-26
Madagascar	434	459	491	520
Malawi	538	574	591	633
Malaysia	44	53	51	60
Maldives	32	40	39	47
Mali	582	596	640	655
Malta	-17	-12	-18	-13
Mauritania	288	308	309	331
Mauritius	-25	-21	-22	-18
Mexico	26	36	26	36
Micronesia (Federated States of)	19	22	17	20
Moldova	-56	-54	-55	-53
Mongolia	65	74	71	81
Montenegro	-31	-29	-29	-27
Morocco	28	34	33	40
Mozambique	470	500	522	557
Myanmar	10	15	20	26
Namibia	176	188	195	209
Nepal	13	19	24	32
Netherlands	2	2	5	5
New Zealand	39	42	38	41
Nicaragua	24	30	27	34
Niger	1326	1391	1464	1541
Nigeria	412	438	449	478
North Korea	0	4	9	15
Norway	59	61	62	63
Occupied Palestinian Territory	319	337	316	333
Oman	89	99	95	106
Pakistan	125	133	141	150

D: **Country/territory level results under all four scenarios.** The numbers display the relative increase in calorie requirements between 2010 and 2100. Each column shows the result for one of the four scenarios.

Country	Stable Weight pct.change	Rising BMI pct. change	Rising Height pct. change	Rising Height & BMI pct. change
Panama	68	75	72	79
Papua New Guinea	177	190	190	204
Paraguay	43	50	48	55
Peru	43	51	47	55
Philippines	86	92	101	108
Poland	-44	-42	-43	-41
Portugal	-31	-28	-30	-27
Puerto Rico	-42	-38	-42	-39
Qatar	62	70	59	67
Romania	-48	-46	-47	-45
Russian Federation	-19	-17	-18	-16
Rwanda	165	177	195	210
Saint Lucia	-4	0	-6	-2
Saint Vincent and the Grenadines	-29	-26	-28	-24
Samoa	53	60	44	50
Sao Tome and Principe	239	256	257	276
Saudi Arabia	72	81	71	80
Senegal	525	545	577	600
Serbia	-42	-41	-41	-40
Seychelles	-14	-12	-12	-10
Sierra Leone	171	185	195	211
Singapore	6	10	13	17
Slovakia	-32	-31	-32	-31
Slovenia	-20	-19	-19	-17
Solomon Islands	176	187	188	200
Somalia	561	598	623	667
South Africa	31	38	33	40
South Korea	-26	-23	-21	-17
South Sudan	353	376	374	400
Spain	-20	-16	-19	-16
Sri Lanka	-27	-24	-21	-17
Sudan	281	301	299	321
Suriname	8	12	9	14
Swaziland	86	95	91	101
Sweden	53	54	56	57
Switzerland	41	44	45	48
Syria	93	104	93	103
Taiwan	-49	-46	-46	-43
Tajikistan	156	167	166	179
Tanzania	610	645	665	704
Thailand	-40	-36	-36	-31
Timor-Leste	222	238	262	282
Togo	368	391	404	431

D: **Country/territory level results under all four scenarios.** The numbers display the relative increase in calorie requirements between 2010 and 2100. Each column shows the result for one of the four scenarios.

Country	Stable Weight pct.change	Rising BMI pct. change	Rising Height pct. change	Rising Height & BMI pct. change
Tonga	66	64	57	55
Trinidad and Tobago	-26	-24	-26	-24
Tunisia	17	21	21	25
Turkey	23	29	23	29
Turkmenistan	14	19	17	21
Uganda	571	604	635	674
Ukraine	-43	-42	-42	-41
United Arab Emirates	44	51	44	51
United Kingdom	30	33	31	34
United States of America	44	46	41	44
Uruguay	-2	1	-2	2
Uzbekistan	19	24	22	28
Vanuatu	205	213	215	223
Venezuela	46	54	48	57
Vietnam	16	21	30	36
West Sahara	103	113	112	122
Yemen	132	145	149	163
Zambia	715	755	786	833
Zimbabwe	209	222	232	247
