# Supplementary figures and images for: The effect of bigger human bodies on the future global calorie requirements
Source: PLoS One. 2019 Dec 4;14(12):e0223188. doi: 10.1371/journal.pone.0223188 (PMC6892500; doi:10.1371/journal.pone.0223188)

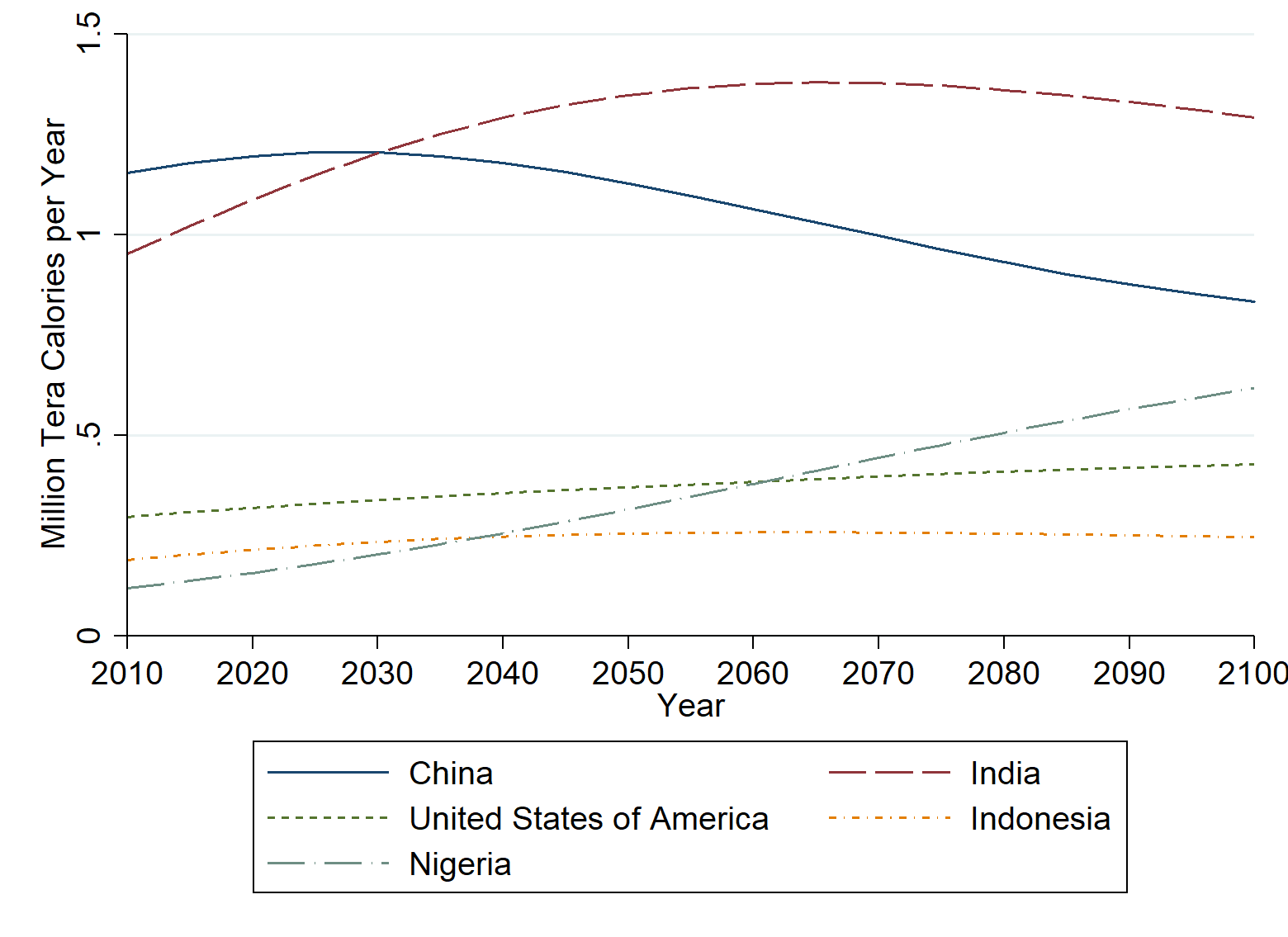

Supplement: S1 Fig — (TIF) [file pone.0223188.s002.tif]

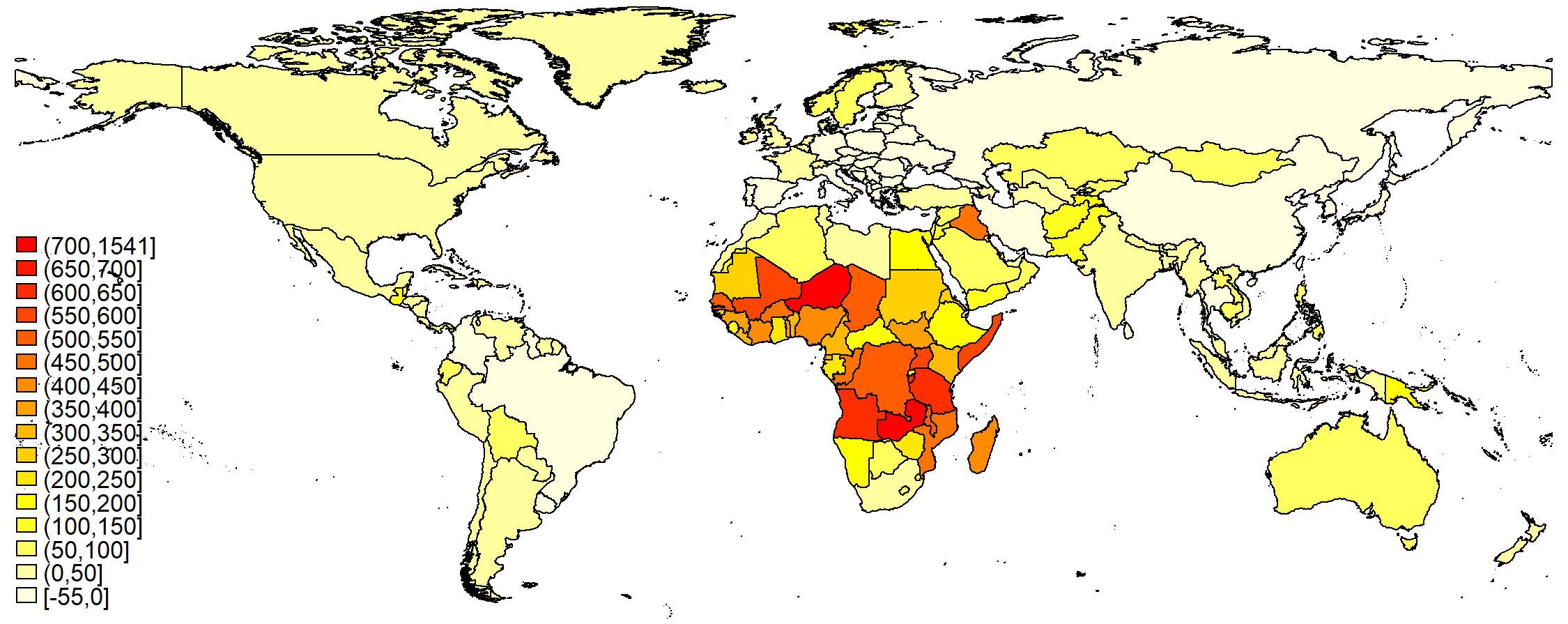

Supplement: S2 Fig — (TIF) [file pone.0223188.s003.tif]
